# Integrative and Complementary Interventions Result in Significant Positive Effect Sizes in Quality of Life and Symptom Burden among Patients with Pediatric Cancer and Other Serious Illness

**DOI:** 10.21203/rs.3.rs-7208100/v1

**Published:** 2025-09-10

**Authors:** Jennifer L. Raybin, Kathleen E. Montgomery, Micah Skeens, Norah Janosy, Scott Mist, Heather Franklin, Nathan F Dieckmann, Verna L. Hendricks-Ferguson, Catherine M. Jankowski

**Affiliations:** University of Colorado College of Nursing and School of Medicine (Drs. Jankowski and Janosy); Saint Louis University School of Nursing (Dr. Hendricks-Ferguson); Oregon Health and Sciences University Schools of Nursing and Medicine (Drs. Raybin, Mist, Franklin, and Dieckmann); University of Wisconsin-Madison School of Nursing (Dr. Montgomery); College of Medicine, The Ohio State University

**Keywords:** integrative, complementary, quality of life, symptoms, childhood cancer, creative arts

## Abstract

**Purpose:**

Children with cancer report intractable symptoms and endorse using complementary and integrative health interventions (CHI) without strong published evidence. We conducted a prospective study of CHI in 100 participants at two children’s hospitals. The secondary aim was to estimate outcome effect sizes. Here we report preliminary effect size estimates of CHI on quality of life (QOL) and symptom burden in pediatric participants with cancer and other serious illness.

**Methods:**

We used standardized patient reported outcome (PRO) measures in response to CHI sessions. Data were collected at baseline, pre/post CHI, and monthly up to 6 months. Outcomes were QOL and symptom burden.

**Results:**

Participants (n=100) were aged 2–29 years (M=13.5, SD=5.6), 65% identified as female, 23% were from underrepresented populations, 52% were receiving treatment for cancer versus other serious illness. Participants completed 191 CHI sessions with 811 PRO assessments. CHI included acupuncture (39%), aromatherapy (35%), creative arts (20%), massage therapy (5%), and hypnosis (1%). Mixed-effects models controlling for cancer diagnosis revealed improvement in anxiety, fatigue, pain, sadness, and mood (Cohen’s d effect sizes 0.20–1.45; all p<0.05). Creative arts were associated with improvement in all symptoms except for nausea (0.31–1.45; all p<0.03). QOL improved over time (b = 0.21; p<0.05) and was clinically significant compared to standard clinical cut offs.

**Conclusion:**

Patients (or proxy) reported improvement in QOL and symptom burden with CHI. The positive effect size estimates support for the need for additional efficacy studies of targeted CHI among children with serious illnesses including cancer, especially creative arts interventions.

Because the cure rate for childhood cancer has improved to over 80% [1], more children are living longer and experiencing symptom suffering. Children with cancer and their family caregivers report using complementary health interventions (CHI) to treat adverse symptoms and address well-being [2–4]. Researchers have reported safety, improved symptom burden, and improved quality of life (QOL) with CHI among children with cancer, but the evidence lacks rigor [2, 5–8]. Initial feasibility and efficacy of CHI interventions in pediatric cancer have been demonstrated for acupuncture [9–11], aromatherapy [12, 13], hypnosis [14], massage therapy, and creative arts therapy [15, 16]. Indeed, we showed an improvement in QOL and reduction in anxiety over time with creative arts therapy among children with cancer, but our findings were also limited by small sample size and historical outcome measures (PedsQL instead of PROMIS) [16, 23]. Although the evidence in support of CHI is mounting, small sample sizes and lack of reproducibility are ongoing themes in the extant literature [17–21].

We therefore conducted a two-site prospective feasibility study of CHI with standardized outcome measures from the NIH toolbox and an accrual goal of 100 participants [22]. We found that CHI was feasible and demonstrated an 87% completion of at least one CHI and two outcome surveys [22]. The 94% accrual rate and 96% acceptance rate reflected interest and popularity of CHI and lack of participant burden in completing the measures. The purpose of this paper is to report the results of the secondary aim: to estimate effect sizes of CHI on QOL and the five priority symptoms of pain, anxiety, nausea, fatigue, and mood with respect to disease status (cancer versus non-cancer). Effect sizes in the expected direction will provide additional support for future efficacy-focused randomized controlled trials.

The theoretical basis for this study included the Obesity-Related Behavioral Intervention Trials (ORBIT) consortium model [24], the Symptom Management Theory (SMT) [25, 26], and the concept of emotional embodiment (QOL is reflected by the physical body) [27–29]. The study was informed by our preliminary work implementing CHI and using rigorous outcome measures in children and adolescents with cancer [16, 30–33]. We purposefully recruited participants with any serious illness to assess feasibility in all pediatric patients but collected diagnosis as a variable to examine initial effect sizes controlling for cancer as a covariate.

## Methods

The study methods were reported in detail with the findings of the primary feasibility aim previously [22]. In brief, the two-site prospective feasibility clinical trial of CHI (ClinicalTrials.gov#: NCT05594693) was conducted at tertiary children’s hospitals at academic medical centers. This study was performed in line with the principles of the Declaration of Helsinki. Approval was granted by the Ethics Committee of University of Colorado Multiple Institutional Review Board (March 8, 2022/No. 21–3665). Approval at the second site was granted by the Oregon Health and Science University Institutional Review Board (March 10, 2023/No. 00020254). We used convenience sampling to recruit participants interested in CHI. Inclusion criteria were children aged from 31 days to 30 years, receiving CHI, and able to self-complete or caregiver proxy complete the surveys. Participants were not required to have a threshold symptom status, but only the desire to engage in CHI. The CHI available were acupuncture, aromatherapy, massage therapy, hypnosis, and creative arts interventions (making visual art with hospital-based art teachers). The interventions were provided by licensed, credentialed interventionists at varying schedules for 30–60 minutes in a convenient location where the patient was receiving usual care (inpatient room, clinic room, infusion bed). Patients who were interested in receiving a CHI were recruited to join the study. Parent/caregivers completed consent and child participants completed age-appropriate assent. Using the Research Electronic Data Capture (REDCap) system [34, 35], surveys were delivered to participants electronically. Participants completed a demographics survey (with parent/caregiver help assistance in 10% of the sample). The participants’ QOL was measured by the Pediatric PROMIS-Global Health-7 [36–39]. [40]. The reported minimally clinical important difference (MCID) to reflect a change in QOL on the PROMIS is 2 points [41]. The participants’ symptoms were assessed before and after each CHI session with the Ped PRO-CTCAE [42–45]. We used the the Faces Scale developed by McGrath at all time points to evaluate emotional response to stimuli (versus pain alone) [16, 30, 31, 46–48]. The MCID on the Faces Scale has been estimated as one face [49, 50].

Effect sizes (e.g. Cohen’s *d*) were estimated using a mixed-effects modeling approach (MEM) including pre/post outcome measures which accounted for nesting within person (i.e., that the same patient could have completed multiple CHI), and controlling for an indicator for cancer diagnosis (versus all other chronic conditions). Cohen’s *d* was defined using the MEM estimated pre/post change in the numerator and the sum of the random error terms from the MEM in the denominator. Cohen’s *d* effect size ranges were classified as small (*d* = *0.2–0.4*), medium (*d* = 0.5–0.7), and large (*d* ≥ 0.8) [51]. We also used mixed-effects models to examine the change in QOL (measured by PROMIS and Faces Scale) over time, with the diagnosis of cancer as a covariate, and compared these changes to the clinically significant change thresholds reported for each tool (2 points on PROMIS and 1 face on Faces Scale).

## Results

The total sample included 100 enrolled participants aged 2–29 years (M=13 years), 64% female (due to the total N=100, only percentages are reported). About half of the participants or caregiver proxies (52%) reported a primary diagnosis as cancer, about a quarter reported chronic pain as the reason for treatment (21%), and the rest of the sample reported a mix of diverse subspecialty disorders (27%) (Table 1), such as neurology, gastroenterology, nephrology, and hematology. The types of cancer recorded were based on self-report, with all three tumor types (solid, liquid, central nervous system) represented (Table 2).

**Figure 4.**
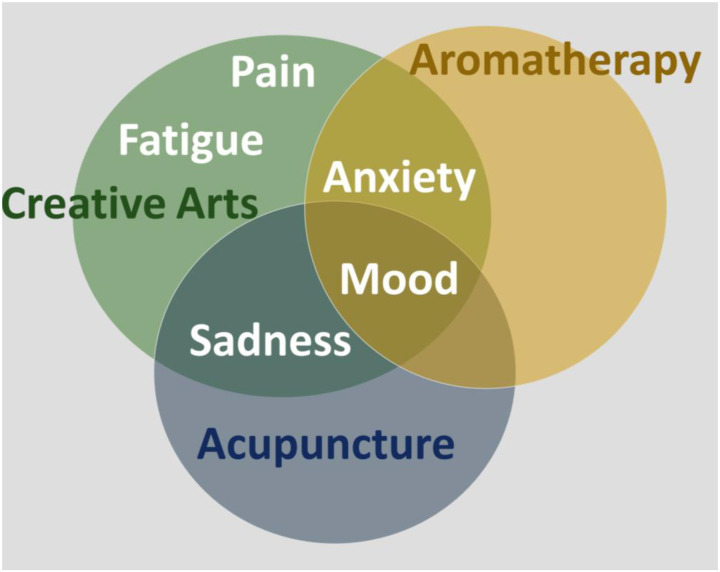
Venn Diagram of Symptoms that Improved with CHI

Because the number of participants varied among the CHI intervention types, we included only symptom data for interventions that were provided 20 times or more to improve statistical precision. The most frequently selected intervention was creative arts therapy, followed by acupuncture and aromatherapy (Table 3). When controlling for the diagnosis of cancer, participants had significant improvements in anxiety, fatigue, pain, sadness, and mood after creative arts with medium-to-large effect sizes (Table 3). Aromatherapy resulted in improved anxiety, pain, sadness, and mood. After acupuncture, participants reported improvement in sadness and mood (Figure 4).

**Figure 5.**
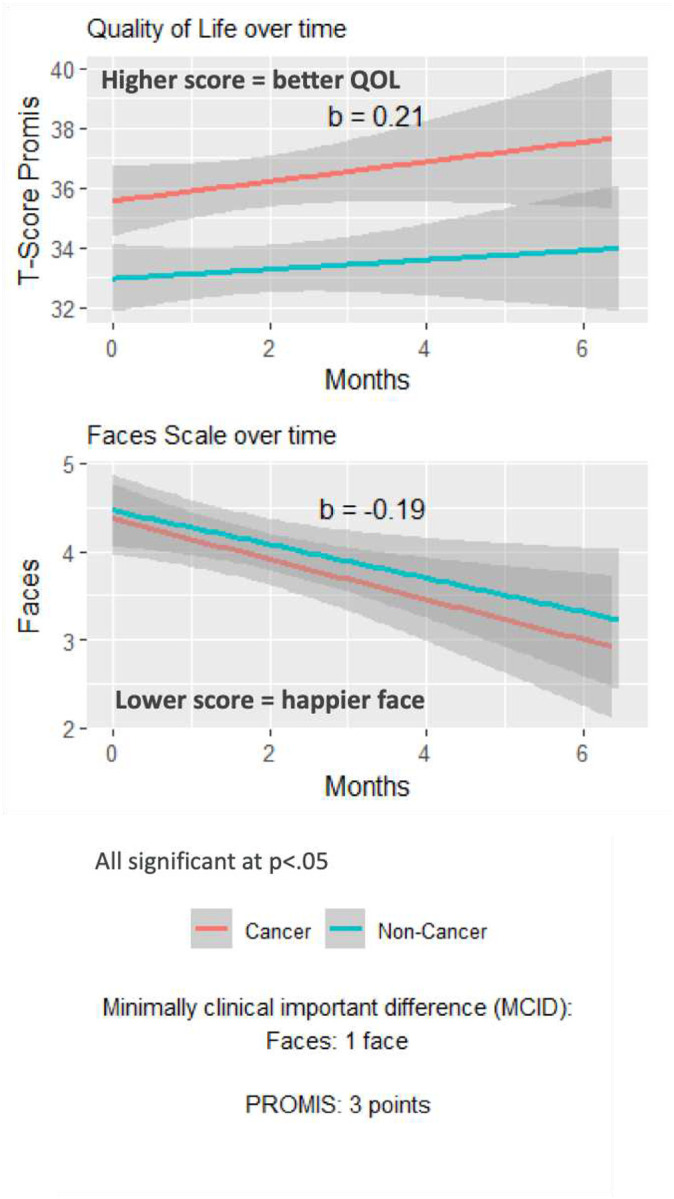
Mixed-effects Regression Models

The participants’ scores on the PROMIS scale improved (b = 0.21; p<0.05) and their scores on the Faces Scale also improved (b = 0.19; p<0.05) over time. Although the rate of change was similar, the participants with cancer started with a better quality of life than those with other serious illnesses (Figure 5). On the PROMIS scale, the magnitude of change was 2 points just above the reported MCID of 2 points. The magnitude of change on the Faces scale was 1.9 faces which surpassed the MCID of 1 face.

## Discussion

Robust statistical and clinical improvement was seen in symptoms and QOL in relation to CHI. These changes were sustained when controlling for the diagnosis of cancer compared to other serious illness. Creative arts interventions were associated with participants recording improvement in four of the five priority symptoms (i.e., pain, anxiety, fatigue, and sadness) as well as improvement in emotional responses on the Faces Scale. Participants who received aromatherapy and acupuncture reported improved emotional response scores on the Faces Scale. As we expected, participants who received aromatherapy and creative arts also showed improvement in anxiety scores. An unexpected finding was improved sadness scores among participants who received acupuncture. Although the study was under-powered to examine the interaction of CHI and cancer, these initial findings support directions for future study in this population and provide a basis for sample size calculations.

The results here replicate some of the findings of our prior studies of creative arts with children with cancer. Our pilot randomized controlled study among 12 children with brain tumors showed improvement in nausea and mood [31], yet in the current study, nausea was not significantly affected by any intervention. Perhaps this was due to the discrepancy in populations. Children with brain tumors are known to experience worse nausea [33] which may have allowed for a more significant improvement compared to this study’s findings. In our subsequent study of creative arts in 100 children with cancer, we demonstrated improvement in QOL and anxiety [23] which was also replicated here. Additionally, the improvements in fatigue, pain, and sadness with creative arts in the current study are promising for further exploration. It is possible that creative arts therapy could join physical activity and mindfulness as evidence-based interventions that improve fatigue in this population [52]. If creative arts could truly improve intractable pain, even in a selected population of children with cancer, this could be a supplementary intervention to augment pain medications, without additional adverse effects. Finally, sadness and mood are symptoms that could affect all aspects of a child’s experience of cancer treatment. By participating in the creative process, a child could feel happier which may improve tolerance of treatment [53].

The results of improved anxiety and mood with aromatherapy in this study are also consistent with research evident in other studies [12, 13, 54]. Although others have demonstrated an improvement in nausea, we did not find any intervention that was associated with improved nausea. Other researchers have documented the difficulty in studying and treating nausea with CHI in children with cancer [55, 56].

Although many pediatric patients with cancer report using acupuncture in the community, our experience in this trial was that oncologists were nervous to allow needle interventions in their patients because of concerns about infection risk. We addressed this hesitancy with provider-focused education modules and journal clubs about the safety of acupuncture in this population [5, 11]. Interestingly, acupuncture was only associated with improvement in sadness and mood in this study. Other studies have found decreased pain and nausea with acupuncture [9, 11, 54]. Perhaps the relaxation in the acupuncture session or the attention of the interventionist helped the mood of the patient which in turn could affect other symptoms.

Despite use of the convenience sampling, this study accrued almost equal groups of participants with and without cancer. We therefore had adequate group sizes to enable comparison of QOL and Faces Scale scores between these two groups over time. All participant scores improved significantly over time. Participants with cancer had more positive (happier) scores at baseline than those with other serious illness, and that difference persisted over time. We could not compare the interactions of specific CHI by cancer due to the limited sample size within each type of CHI. Others have found that children with cancer report feeling better as they progress through therapy and treatment [57], although severe, debilitating symptoms occur during advanced cancer [33, 58]. Therefore, we propose that creative arts and aromatherapy, with limited invasiveness and medium to large effect sizes, may be well-suited for use in children with advanced cancer.

## Limitations

This study was not powered for efficacy; thus, all results must be viewed as preliminary signals for future exploration with adequate rigor. The number of massage therapy sessions did not reach our threshold of 20, so we did not evaluate effect size. The wide inclusion criteria allowed for a heterogenous population which limits specificity. Because of the variability in the number of CHI sessions received, these data are influenced by level of exposure to CHI. We did not collect demographic data on the caregivers who completed 10% of the child survey responses by proxy.

## Conclusions

Pediatric patients with cancer and other chronic conditions benefitted from CHI. Creative arts interventions continue to show promise as helpful and noninvasive activities to improve physical (fatigue, pain) and psychologic (anxiety, mood) symptoms. Hospital approved aromatherapy showed benefit as a low risk intervention for psychologic symptoms (anxiety and mood). In this study, acupuncture was related to improved sadness. These interventions are not likely to cause adverse interactions. We now have the effect sizes with which to power a multi-site efficacy randomized controlled trial. Rigorous studies should be prioritized to continue to improve children’s experience of cancer therapy.

## Figures and Tables

**Figure 1.**
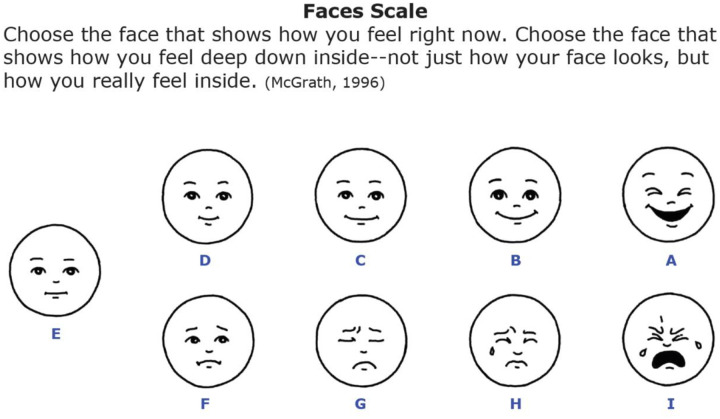
Faces Scale

**Figure 2.**
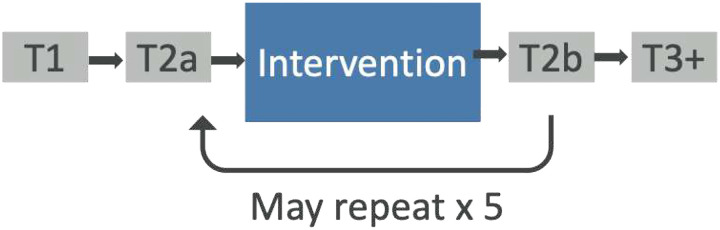
Study Schema

**Figure 3.**
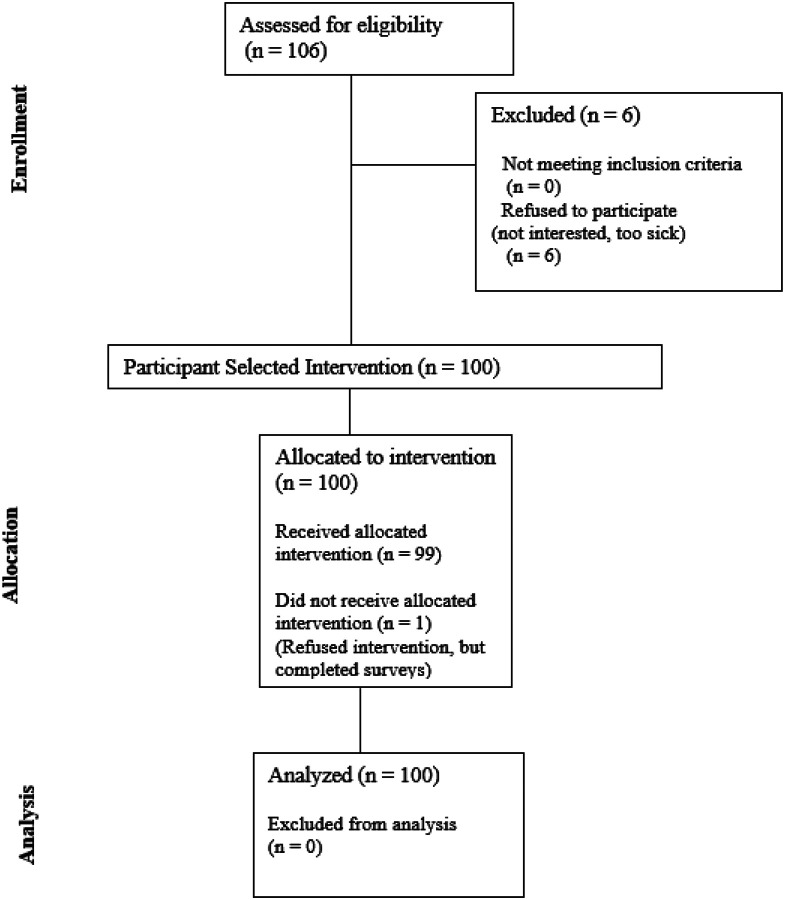
Study Flow Diagram

**Table 1.** Demographics

	Overall (N=100)
**Age**	
Mean (SD)	13.3 (5.54)
Median [Min, Max]	14.0 [2.00, 29.0]
**Diagnosis**	
Cancer	52
Non-Cancer	48
**Gender**	
Female	64
Male	31
Missing	5
**Race**	
White/Caucasian	75
Black/African American	4
Asian/Native Hawaiian/Other Pacific Islander	0
Native American/Alaska Native	0
More than One Race	11
Other	5
Unknown/Not Reported/Missing	9
**Ethnicity**	
Hispanic	20
Non-Hispanic	76
Unknown/Not Reported/Missing	4
**Parent Education**	
No College	23
Some to Complete College	41
Graduate School	29
Decline to Answer or Missing	7

Note: N = number of participants, SD = standard deviation. Due to the total N=100, percentages are equivalent to the raw number listed.

**Table 2.** Patient Reported Specific Cancer Type

Disease Type	Diagnostic Category	Participant Free-Text Description	N
Cancer			51
	Hematologic Malignancy	Leukemia Lymphoma	15
	Solid Tumor	Neuroblastoma Osteosarcoma Langerhans Cell Histiocytosis Malignant Pleural Effusion	11
	Central Nervous System Tumor	Diffuse Midline Glioma Pilocytic Astrocytoma Intracranial Germ Cell Tumor	4
	Bone Marrow Transplant	Severe Aplastic Anemia NOS	2
	NOS		19

Note: NOS = not otherwise specified; N = number of participants

**Table 3.** Significant Symptom Changes after Interventions: Coefficients from mixed effects models, controlling for diagnosis of cancer

Symptom	Acupuncture (155 obs, n=33)	Aromatherapy (86 obs, n=20)	Creative Arts (134 obs, n=38)
**Anxious**			
Frequency		−0.35 (p = 0.01)	−0.42 (p = 0.01)
**Fatigue**			
Frequency			−0.39 (p = 0.03)
**Pain**			
Frequency		−0.24 (p = 0.03)	−0.37 (p = 0.01)
**Sadness**			
Frequency	−0.20 (p = 0.02)	−0.37 (p = 0.01)	−0.31 (p = 0.03)
Severity	−0.46 (p = 0.05)		
Interference			−0.44 (p = 0.02)
**Mood**			
Frequency	−0.92 (p = 0.01)	−0.71 (p = 0.04)	−1.45 (p = 0.01)

Note: obs = number of touch points; n = number of CHI sessions; Mood was measured by the Faces Scale; Frequency = how often; Interference = interferes with daily activities. Effect sizes were estimated using mixed-effects models including pre-post outcome measures to account for within-person nesting.
